# Codocytosis in the Dog: 345 Cases (2020–2022)

**DOI:** 10.1111/vcp.70107

**Published:** 2026-04-15

**Authors:** Sarena M. Krojanker, Maria Jaramillo, Brad D. Cingolani, Floyd L. Williams, Priscila B. S. Serpa, Andrew D. Woolcock

**Affiliations:** ^1^ Department of Veterinary Clinical Sciences, College of Veterinary Medicine Purdue University West Lafayette Indiana USA; ^2^ Department of Population Medicine and Diagnostic Sciences, College of Veterinary Medicine Cornell University Ithaca New York USA

**Keywords:** blood smear analysis, erythrocyte morphology, target cells

## Abstract

**Introduction:**

Codocytes, or target cells, are a morphologic variation of erythrocytes characterized by increased membrane surface area relative to volume. In dogs, codocytosis is frequently noted on blood smear evaluation, but its clinical significance remains poorly understood.

**Objectives:**

To characterize the clinical conditions associated with codocytosis in dogs and assess relationships between codocyte severity and hematologic, biochemical, and diagnostic findings.

**Methods:**

A retrospective descriptive study was conducted on 345 dogs from a veterinary teaching hospital from September 2020 through October 2022. Dogs were included if their CBC report described codocytosis graded as ≥ 2+. Data collected included CBC results, serum biochemistry, clinical diagnoses, and current medications. Codocytosis was graded semi‐quantitatively (2+ to 4+). Statistical comparisons were made using chi‐square tests.

**Results:**

Codocytosis ≥ 2+ was identified in 2.5% of 14 010 canine CBCs during the study. The majority (81.4%) had 2+ codocytes. There were no significant differences in anemia, macrocytosis, or regeneration across grades. Dogs with 3+ or 4+ codocytosis had significantly higher alanine transferase (ALT) activity and were more frequently diagnosed with hepatobiliary (*p* = 0.006) and gastrointestinal (*p* = 0.009) diseases. Endocrine disease, particularly diabetes mellitus was also frequently observed. Antibiotic use was significantly associated with higher codocyte grades (*p* < 0.001), though no causation was identified.

**Conclusions:**

Codocytosis in dogs is not primarily associated with anemia or regeneration, but rather appears more frequently in diseases affecting hepatobiliary, gastrointestinal, and endocrine systems. These findings suggest a multifactorial pathogenesis likely involving altered lipid metabolism and membrane remodeling. Recognition of codocytosis may provide insight into systemic disease processes in canine.

## 1 Introduction

Target cells, or codocytes, are bell‐shaped erythrocytes with a bullseye appearance, which results from an increased surface area‐to‐volume ratio, often due to membrane excess or decreased intracellular content, and is associated with reduced osmotic fragility [1, 2].

In dogs, codocytes are commonly described on peripheral blood smears, yet their diagnostic and clinical significance remains unclear. Proposed associations with codocytosis are largely extrapolated from human medicine, where codocytes have been linked to regenerative anemia, liver disease, hemoglobinopathies, and postsplenectomy states [3, 4, 5, 6]. In regenerative anemia, immature erythrocytes possess excess membrane, which predisposes them to codocyte morphology during smear preparation. In addition, macrocytic erythrocytes with weaker membranes may be more prone to deformation, enhancing the codocyte appearance [7, 8].

Codocytosis may also occur in non‐anemic states. In hepatic disorders, altered lipid metabolism—particularly increased cholesterol‐to‐phospholipid ratios—can modify erythrocyte membranes and promote codocyte formation [9, 10, 11, 12]. Similar changes may occur in hypothyroidism, which also affects lipid handling. Congestive heart failure has been associated with codocytosis through multifactorial mechanisms, including chronic hypoxia and erythropoietin‐driven ineffective erythropoiesis, hepatic congestion impairing lipid metabolism, and diminished splenic filtration of poikilocytes [13]. Post‐splenectomy codocytosis is attributed to loss of erythrocyte culling and metabolic alterations affecting membrane composition [8].

In a recent study of 312 diabetic dogs, codocytosis was observed more frequently in patients with diabetic ketoacidosis, potentially due to disrupted lipid metabolism and oxidative injury [14]. Despite these observations, the incidence, disease associations, and diagnostic relevance of codocytosis in dogs have not been investigated.

The objective of this retrospective, descriptive study was to characterize clinical conditions associated with codocytosis in dogs by evaluating hematologic profiles, clinical diagnoses, and pharmacologic histories. Improved understanding of codocyte‐associated morbidities may enhance the interpretation of blood smears and inform clinical decision‐making in veterinary patients.

## 2 Materials and Methods

### 2.1 Study Design and Case Selection

This retrospective descriptive study was conducted at a single veterinary teaching hospital. The study period spanned from September 2020 through October 2022. Electronic medical records were searched using the hospital's laboratory information system to identify canine patients with CBCs that included a reported RBC morphology of codocytosis graded as ≥ 2+.

Codocytosis grading was derived from microscopic peripheral blood smear evaluation performed by board‐certified clinical pathologists. Dogs were included if codocytosis was graded as 2+ (moderate) or greater; patients with < 2+ codocytosis were excluded from descriptive and statistical analyses.

### 2.2 Data Collection

The following data were extracted for each dog: signalment (age, breed, sex), pre‐existing and current medical conditions, current medications, and results of CBC, serum biochemistry, and peripheral blood smear evaluation. Diagnoses were categorized as either pre‐existing (established before the visit associated with codocytosis) or new (diagnosed at the time of codocytosis detection). Disease categories included cardiac, endocrine, gastrointestinal/pancreatic, hepatobiliary, immune‐mediated, infectious, musculoskeletal, neoplasia, neurological, ophthalmic, renal/urinary, and other. It was also documented if dogs had diagnoses in multiple categories. Infectious diagnoses were categorized independently unless they involved a region covered by another specific category (e.g., cholangiohepatitis classified under hepatobiliary). Diagnoses were made and coded by the attending clinicians and documented for the study through review of the medical records.

Medications administered at the time of codocytosis detection were reviewed and categorized by pharmacologic class. These included: antibiotics, anticonvulsants, cardiac medications (e.g., angiotensin converting enzyme inhibitors, angiotensin receptor blockers, diuretics, pimobendan, antiarrhythmics, anticoagulants), chemotherapy/immunomodulators (e.g., cyclophosphamide, vincristine, mycophenolate, azathioprine, cyclosporine), corticosteroids, gastrointestinal agents (e.g., antiemetics, prokinetics, proton pump inhibitors), non‐steroidal anti‐inflammatory drugs, and opioids.

Each drug category was defined based on the known primary mechanism of action and/or body system for which its use is primarily selected. Combination therapies were recorded in multiple relevant categories.

### 2.3 Hematology, Blood Smear Evaluation, and Serum Chemistry Profiles

CBCs were performed using the ADVIA 2120i Hematology System (Siemens Healthcare Diagnostics, Tarrytown, NY, USA). Manual leukocyte differential counts and RBC morphology evaluations were performed by a medical technologist and reviewed by a clinical pathology resident and a board‐certified clinical pathologist on Modified Wright‐stained blood smears. Blood smears were prepared using fresh ethylenediaminetetraacetic acid anticoagulated whole blood and stained using an automated slide stainer (Hematek 3000, Siemens Healthcare Diagnostics).

RBC morphologic abnormalities were recorded, including codocytes, acanthocytes, dacryocytes, echinocytes, elliptocytes, keratocytes, ghost cells, spherocytes, stomatocytes, nucleated RBCs, reticulocytes, schistocytes, eccentrocytes, and Howell‐Jolly bodies. RBC characteristics such as anisocytosis, macrocytosis, microcytosis, poikilocytosis, polychromasia, and rouleaux were also recorded. Inclusions such as basophilic stippling, Heinz bodies, and Howell‐Jolly bodies were noted when present.

Codocytosis was graded using a semi‐quantitative system adapted from Weiss et al., based on counts performed at 1000× magnification using an oil immersion objective [15]. The average number of codocytes was determined from ten uniform microscopic fields, each containing approximately 250–300 RBCs. The grading scheme used for codocytes is depicted in Table 1.

**TABLE 1:** Grading scale for codocyte identification on blood smear evaluation as established by the clinical pathology laboratory at Cornell University Veterinary.

Severity of codocyte identification	Grade	Mean # of codocytes across 10 hpf
Mild	1+	3–5
Moderate	2+	6–15
Marked	3+	16–30
Severe	4+	> 30

*Note:* Only dogs with moderate, marked, or severe codocytosis were included in the study.

Only dogs with ≥ 2+ codocytosis (i.e., a mean codocyte count of ≥ 6 cells per field) were included for further analysis. Other RBC abnormalities were graded according to the established guidelines of the American Society for Veterinary Clinical Pathology and as described by Weiss et al. [15, 16].

Total WBC counts and differential cell counts (segmented neutrophils, band neutrophils, lymphocytes, monocytes, eosinophils, and basophils) were recorded. Neutrophil toxic changes (Döhle bodies, toxic granulation, vacuolation) and the presence of hypersegmented neutrophils were noted. Platelet counts were recorded, and platelet aggregation was noted when present.

Serum biochemistry profiles were performed using the Cobas c501 analyzer (Roche Diagnostics, Indianapolis, IN, USA) with manufacturer‐supplied reagents. Analytes included: glucose, blood urea nitrogen (BUN), creatinine, phosphorus, calcium, sodium, potassium, chloride, bicarbonate, anion gap, total protein, albumin, globulin, ALT, alkaline phosphatase (ALP), gamma glutamyl transferase (GGT), total bilirubin, cholesterol, amylase, and lipase.

### 2.4 Statistical Analysis

Comparisons of proportions of categorical data were conducted using the chi‐square test. In analyses, values of *p* < 0.05 were considered statistically significant. Statistical analyses were conducted using commercially available software (Stata v. 16.1, StataCorp LLC, College Station, TX, USA).

## 3 Results

During the study period, 14 010 complete blood counts were performed in dogs. Of these, 907 dogs had codocytes noted on blood smear analysis of their initial CBC (6.5%). Five hundred sixty‐two of these dogs had only 1+ codocytosis noted, so they were excluded from continued analysis. This resulted in 345 dogs included in the study. The incidence of substantial codocytosis in dogs was 2.5% over the 2‐year study period. Within the 345 dogs, 281 were noted to have 2+ codocytosis (C2, 81.4%), 57 dogs had 3+ codocytosis (C3, 16.5%), and 7 dogs had 4+ codocytosis (C4, 2.0%). The demographics of each of these groups are depicted in Table 2. The median age did not differ between groups based on frequency of codocytes (*p* = 0.38).

**TABLE 2:** Signalment data for the dogs stratified by severity of codocytes.

Parameter	C2 (*N* = 281)	C3 (*N* = 57)	C4 (*N* = 7)
Age (median [range])	9 years [0.17–17]	8 years [0.17–16]	10 years [2–15]
Sex (*N*, %)	M = 113, 40.2%	M = 18, 31.6%	M = 1, 14.3%
	MC = 45, 16.0%	MC = 13, 22.8%	MC = 0
	F = 83, 29.5%	F = 14, 24.5%	F = 2, 28.6%
	FS = 40, 14.3%	FS = 12, 21.1%	FS = 4, 57.1%
Breed (*N*, %; listed if > 2%)	Mixed breed (68, 24.2%)	Mixed breed (10, 17.9%)	1 each (14.2%):
	Labrador Ret (26, 9.3%)	Labrador Ret (9, 16.1%)	Aust Shepherd
	Golden Ret (20, 7.1%)	Yorkshire Terrier (4, 7.1%)	Basset Hound
	Shih Tzu (13, 4.6%)	Golden Ret (3, 5.4%)	Dachshund
	American Pitbull (11, 3.9%)	Goldendoodle (3, 5.4%)	Mini Schnauzer
	Yorkshire Terrier (9, 3.2%)	Pug (3, 5.4%)	Mixed breed
	Chihuahua (8, 2.8%)	Cocker Spaniel (2, 3.6%)	Norwegian
	Aust Shepherd (7, 2.5%)	American Pitbull (2, 3.6%)	Elkhound
	Beagle (7, 2.5%)	Chihuahua (2, 3.6%)	Shih Tzu
	Mini Schnauzer (7, 2.5%)		
	German Shepherd (6, 2.1%)		
	Poodle (6, 2.1%)		
	Pug (6, 2.1%)		

*Note:* C2 indicates dogs with moderate (2+) codocytes. C3 indicates dogs with marked (3+) codocytes. C4 indicates dogs with severe (4+) codocytes.

Abbreviations: Aust Shepherd, Australian Shepherd; F, female intact; FS, female spayed; Golden Ret, Golden Retriever; Labrador Ret, Labrador Retriever; M, male intact; MC, male castrated; Mini Schnauzer, Miniature Schnauzer.

When comparing other CBC parameters between groups, there were no significant differences identified. There were similar numbers of dogs classified as anemic (RI: RBC < 5.5 × 10 [6]/μL, Hct < 37.0%) in all groups (C2 130/281, 46.3%; C3 28/57, 49.1%; C4 4/7, 57.1%; *p* = 0.83). There were similar proportions of dogs with macrocytic erythrocytes (RI: MCV > 75) in all groups (C2 45/281, 16.0%; C3 9/57, 15.8%; C4 1/7, 14.3%; *p* = 0.36). There were similar proportions of dogs with reticulocytosis (RI: Reticulocytes > 100 × 10^3^/μL) in all groups (C2 111/281, 39.5%; C3 26/57, 45.6%; C4 2/7, 28.6%; *p* = 0.82). Median values for other CBC parameters are listed in Table 3.

**TABLE 3:** CBC values for each group of dogs, stratified by severity of codocytes and presented as median [range].

Parameter	Ref interval	C2 (*N* = 281)	C3 (*N* = 57)	C4 (*N* = 7)
RBC	6.0–8.0 × 10^6^/μL	5.6 [1.0–9.58]	5.5 [2.9–9.0]	5.6 [2.9–8.7]
Hgb	12.0–18.0 g/dL	12.4 [2.2–18.60]	11.8 [5.7–18.9]	11.6 [10.8–19.2]
MCV	60–75 fL	69.8 [44–108.5]	69.8 [37.4–92.2]	68.8 [61.2–78.4]
MCHC	32–36 g/dL	32.7 [23.6–39.7]	31.9 [27.1–40.8]	32.4 [30.8–36.2]
Hct	37%–55%	37.9 [8.2–63.5]	37 [21–56.8]	35.5 [21–59.2]
Retic	< 100 × 10^3^/μL	80.7 [2.1–497.6]	85.3 [1.8–345.3]	76.5 [17.5–179.9]
WBC	6.0–17.0 × 10^3^/μL	13.8 [0.2–80.8]	14.7 [0.3–44.1]	11.5 [5.7–19.20]
Segs	3.0–12.0 × 10^3^/μL	10.7 [0.1–68.8]	11.4 [0.6–38.4]	9.8 [2.0–20.4]
Bands	< 0.3 × 10^3^/μL	0 [0–12.8]	0 [0–6.0]	0 [0–0.46]
Lymphs	1.0–5.0 × 10^3^/μL	1 [0–66.3]	1.1 [0.2–7.3]	1.3 [0.4–17.4]
Monos	0.1–1.35 × 10^3^/μL	0.8 [0–6.1]	0.9 [0.1–5.1]	0.9 [0.1–3.0]
Eos	0.1–1.25 × 10^3^/μL	0.1 [0–3.7]	0.3 [0–1.4]	0.7 [0–1.7]
Baso	0.1–0.5 × 10^3^/μL	0 [0–0.4]	0 [0–0.4]	0 [0]

*Note:* C2 indicates dogs with moderate (2+) codocytes. C3 indicates dogs with marked (3+) codocytes. C4 indicates dogs with severe (4+) codocytes. No significant differences were noted when comparing values between groups.

Abbreviations: Bands, band neutrophils; Baso, basophils; Eos, eosinophils; Hct, hematrocrit; Hgb, hemoglobin; Lymphs, lymphocytes; MCHC, mean cell hemoglobin concentration; MCV, mean corpuscular volume; Monos, monocytes; Ref interval, reference interval; Retic, reticulocytes; Segs, segmented neutrophils.

When comparing serum chemistry profiles between groups, significant differences were noted in one of the values associated with the hepatobiliary system, but the remainder of the biochemical parameters were not different between groups. Median ALT values were different by group (RI: 3–69 IU/L, *p* < 0.001), with C4 (156 U/L [65–4050]) significantly higher than C2 (91 U/L [2.3–4500], *p* < 0.001) and C3 (118 U/L [21–3180], *p* < 0.001). Median ALT activity was similar between group C2 and group C3 (*p* = 0.87). Median values for other serum biochemistry profile parameters are listed in Table 4.

**TABLE 4:** Serum biochemistry values for each group of dogs, stratified by severity of codocytes and presented as median (range).

Parameter	Ref interval	C2 (*N* = 281)	C3 (*N* = 57)	C4 (*N* = 7)
Glucose	67–132 mg/dL	105 [27–1057]	106 [68–539]	99 [37–107]
BUN	7–32 mg/dL	16 [3–329]	15 [2–77]	12 [3–44]
Creatinine	0.5–1.5 mg/dL	0.8 [0.2–10.1]	0.6 [0.2–4.9]	0.7 [0.4–1.2]
Calcium	9.7–12.3 mg/dL	10.2 [3.8–13.6]	9.9 [6.2–12]	9.8 [8.6–11.5]
Phosphorus	2.2–7.9 mg/dL	4.5 [1.9–16.6]	4.5 [2–10.1]	4.6 [2.9–8.2]
Sodium	138–148 mmol/L	143 [122–182]	143 [116–167]	142 [138–152]
Potassium	3.5–5.0 mmol/L	4.4 [2–6.9]	4.4 [2.4–6.2]	4.3 [3.5–5.2]
Chloride	105–117 mmol/L	112 [96–142]	111 [91–138]	115 [107–118]
Total protein	4.8–6.9 g/dL	6 [2.2–9.1]	5.6 [3.5–8.3]	6 [4–7.1]
Albumin	2.3–3.9 g/dL	3.2 [1.1–5.1]	2.9 [1.4–5.0]	2.7 [1.9–3.7]
Globulin	1.7–3.8 g/dL	2.8 [1.1–5.6]	2.7 [1.4–4.0]	2.8 [2.1–3.4]
ALT	3–69 IU/L	91 [2.3–4500]^a^, ^b^	118 [21–3180]^a^, ^c^	156 [65–4050]^a^, ^b^, ^c^
ALP	20–157 IU/L	265 [0–18 628]	186.5 [26–13 709]	375 [81–6332]
GGT	5–16 IU/L	13 [0–1279]	12 [0–398]	20 [10–255]
Total bilirubin	0.1–0.8 mg/dL	0.3 [0–25.6]	0.3 [0–20.2]	0.3 [0–17.8]
Cholesterol	125–301 mg/dL	212.5 [0–762]	198.5 [71–884]	206 [51–820]

*Note:* C2 indicates dogs with moderate (2+) codocytes. C3 indicates dogs with marked (3+) codocytes. C4 indicates dogs with severe (4+) codocytes.

Abbreviations: ALP, alkaline phosphatase; ALT, alanine transferase; BUN, blood urea nitrogen; GGT, gamma glutamyl transferase; Ref Interval, reference interval.

^a^ Significant difference when comparing C2, C3, and C4.

^b^ Significant difference when comparing C2 and C4.

^c^ Significant difference when comparing C3 and C4.

Five hundred twenty‐nine diagnoses were recorded in the 345 dogs enrolled in the study. One hundred forty dogs had multiple diagnoses. The most common diagnosis amongst all dogs and all recorded diagnoses was neoplasia (*N* = 115; 33.3% of dogs, 21.7% of diagnoses). Hepatobiliary diseases (*N* = 65; 18.8% of dogs, 12.3% of diagnoses), gastrointestinal/pancreatic diseases (*N* = 58; 16.8% of dogs, 11.0% of diagnoses), and infectious diseases (*N* = 51; 14.8% of dogs, 9.6% of diagnoses) were also common amongst dogs with codocytes of all frequencies. The distribution of all diagnoses is depicted in Table 5. When compared across frequency of codocytes, the distribution was significant amongst hepatobiliary diseases (*p* = 0.006) and gastrointestinal diseases (*p* = 0.009), with higher proportions of dogs diagnosed with diseases of these body systems in C3 and C4 compared to C2.

**TABLE 5:** Distribution of 529 diagnoses in 345 dogs with codocytosis, stratified by severity of codocytes and presented as number, percentage.

Body system	All (*N* = 345)	C2 (*N* = 281)	C3 (*N* = 57)	C4 (*N* = 7)	Common diagnoses
Cardiac diseases	27, 7.8%	24, 8.5%	3, 5.3%	0	Myxomatous mitral valve disease (18), uncharacterized heart murmur (14)
Endocrine diseases	32, 9.3%	26, 9.3%	6, 10.5%	0	Diabetic ketoacidosis (16), diabetes mellitus (12)
Gastrointestinal/Pancreatic diseases	54, 15.7%	44, 15.7%^a^	7, 12.3%^a^	4, 57.1%^a^	Pancreatitis (21), Chronic enteropathy (12)
Hepatobiliary diseases	59, 17.1%	42, 14.9%^a^	13, 22.8%^a^	4, 57.1%^a^	Portosystemic shunt (28), gallbladder mucocele (8)
Immune—mediated diseases	7, 2.0%	4, 1.4%	3, 5.3%	0	Immune–mediated thrombocytopenia (4)
Infectious diseases	45, 13.0%	39, 13.9%	6, 10.5%	0	Skin infections (11), sepsis (10), Parvovirus (10)
Musculoskeletal diseases	6, 1.7%	6, 2.1%	0	0	Fractures (4), stifle disease (3)
Neoplasia	102, 29.6%	79, 28.1%	22, 38.6%	1, 14.3%	Lymphoma/leukemia (28), hemangiosarcoma (19), mast cell tumor (8)
Neurological diseases	23, 6.7%	22, 7.8%	1, 1.8%	0	Seizure disorders (6), spinal cord injury (6)
Ophthalmic diseases	11, 3.2%	10, 3.6%	0	1, 14.3%	Cataracts (3), keratoconjunctivitis sicca (2)
Renal/Urinary diseases	37, 10.7%	33, 11.7%	4, 7.0%	0	Nephrourocystoliths (13), urinary tract infection (12), acute kidney injury (7)
Other	60, 17.4%	49, 17.45	11, 19.3%	0	

*Note:* C2 indicates dogs with moderate (2+) codocytes. C3 indicates dogs with marked (3+) codocytes. C4 indicates dogs with severe (4+) codocytes. Numbers of dogs in each broad category (All, C2, C3, C4) are listed in the column heading, but the total number of dogs listed in each column sums to a greater number than the heading due to 140 dogs having multiple diagnoses.

^a^ Indicates a significant difference in the proportions between groups.

Neoplasia was diagnosed in 102 dogs and of those 28 (24.3%) were diagnosed with lymphoma or leukemia. Other common tumors were hemangiosarcoma (*N* = 19 dogs, 16.5%) and mast cell tumor (*N* = 8 dogs, 7.0%). Gastrointestinal/pancreatic diseases were diagnosed in 54 dogs, and of those, 21 (36.2%) were diagnosed with or suspected to have pancreatitis. Chronic enteropathies, including inflammatory bowel disease, lymphangiectasia, and protein‐losing enteropathy, were also common in this group (*N* = 12 dogs, 20.7%). Infectious diseases were diagnosed in 45 dogs with dermatologic infections, such as pyoderma and otitis, diagnosed in 11 dogs (21.6%). Other common infections in this group included septic processes such as pyothorax and septic peritonitis (*N* = 10 dogs, 19.6%), as well as canine Parvoviral enteritis (*N* = 10, 19.6%).

Hepatobiliary diseases were diagnosed in 59 dogs with portosystemic shunts or other congenital abnormalities, being most common in this group in 28 dogs (43.1%). Gallbladder mucocele was another common diagnosis in 8 dogs (12.3%). The median age of dogs diagnosed with hepatobiliary disease (6 years, range 0.25–14 years) was significantly lower when compared to the whole population of dogs (*p* = 0.005).

While endocrine diseases were not one of the most common body systems represented in dogs with codocytes (*N* = 32, 9.3%), 28 of the dogs diagnosed with endocrine diseases were diagnosed with diabetes mellitus with or without ketoacidosis. Of the 26 dogs in C2 with endocrine disease, 22 were diabetic dogs (10 with diabetes mellitus and 12 with diabetic ketoacidosis). All 6 dogs in C3 with endocrine disease were diabetics (2 with diabetes mellitus and 4 with diabetic ketoacidosis).

One hundred four dogs were not receiving any medications on the day of blood collection for the CBC, on which codocytes were noted. Two hundred forty‐one dogs were receiving at least one medication. Sixty dogs were receiving one medication (17.4%), and 181 dogs were receiving more than one medication (52.5%). Antibiotic medications were the most common in dogs with codocytes (*N* = 127, 36.8%). When comparing the frequency of codocytes, the distribution of antibiotic usage was significant, with a higher proportion of dogs receiving antibiotics in C3 and C4 compared to C2 (*p* < 0.001). Anti‐emetics (*N* = 114, 33.0%; *p* = 0.26) and corticosteroids (*N* = 75, 21.7%; *p* = 0.28) were other common medications in these dogs, but were not significantly distributed across the severity of codocytosis. The most common antibiotics described in dogs with codocytosis were metronidazole (*N* = 46/127, 36.2%), penicillins (*N* = 38/127, 29.9%), fluoroquinolones (*N* = 14/127, 11.0%), and doxycycline (*N* = 13/127, 10.2%). Thirteen dogs (10.2%) were receiving multiple antibiotics. Medications are summarized in Table 6.

**TABLE 6:** Distribution of medication usage in the 345 dogs with codocytosis, stratified by severity of codocytes and presented as number, percentage.

Medication	All (*N* = 345)	C2 (*N* = 281)	C3 (*N* = 57)	C4 (*N* = 7)	Common medications
Antibiotics	127, 36.8%	106, 37.7%^a^	17, 29.8%^a^	4, 57.1%^a^	Metronidazole (46), penicillin (38), fluoroquinolone (14), doxycycline (13), cephalosporine (10), neomycin (9)
Anticonvulsants	81, 23.5%	73, 26.0%	7, 12.3%	1, 14.3%	Gabapentin (59), levetiracetam (19)
Antiemetics/GI support	114, 33.0%	99, 35.2%	14, 24.6%	1, 14.3%	Maropitant (56), omeprazole (50), ondansetron (27)
Cardiac	41, 11.9%	38, 13.5%	3, 5.3%	0	Antithrombotic (14), diuretic (12), antihypertensive (10), ACE inhibitor (9)
Chemotherapy/Immunosupp.	42, 12.2%	35, 12.5%	7, 12.3%	0	Vinka alkaloids (8), mycophenolate (8), palladia (7), chlorambucil (7), doxorubicin (5)
Corticosteroids	75, 21.7%	65, 23.1%	9, 15.8%	1, 14.3%	Prednisone (67), prednisolone (7), budesonide (1)
NSAID	35, 10.1%	33, 11.7%	2, 3.5%	0	Carprofen (12), grapiprant (9), meloxicam (8), piroxicam (5)
Opioids	32, 9.3%	29, 10.3%	3, 5.3%	0	Tramadol (14), codeine (8), fentanyl patch (5)

*Note:* C2 indicates dogs with moderate (2+) codocytes. C3 indicates dogs with marked (3+) codocytes. C4 indicates dogs with severe (4+) codocytes.

Abbreviations: ACE, angiotensin converting enzyme; GI, gastrointestinal; Immunosupp, immunosuppressive; NSAID, non‐steroidal anti‐inflammatory drug.

^a^ Indicates a significant difference in the proportions between groups.

## 4 Discussion

Codocytes were identified in about 6.5% of all canine blood smears from a specialty hospital population, with 2.5% being noted at frequencies of 2+ or higher. To the authors' knowledge, this is the first report of codocytosis prevalence in a canine population. This prevalence is consistent with the general perception of codocytes as a relatively uncommon but recognizable poikilocyte type in dogs. Importantly, this is the first study to characterize the clinical contexts and disease associations of codocytosis in dogs, providing insight into its potential diagnostic and pathophysiologic relevance.

Despite prior suggestions that codocytes are primarily associated with regenerative anemia or erythropoietic disorders, our findings indicate that codocytosis in dogs is not strongly correlated with anemia, macrocytosis, or regenerative response [17, 18]. Similar proportions of anemic and macrocytic dogs were observed across all codocyte grades, and reticulocytosis did not differ significantly among groups. These findings suggest that while codocytosis may accompany regenerative responses, it is not exclusive to anemic states and may be driven by alternative mechanisms such as membrane lipid remodeling. A previous description of dyserythropoiesis, polymyopathy, and cardiac disease in three English springer spaniels reported codocytes as a common finding, but no patients of this breed or this combination of problems were identified in the current study; however, it is possible that cases of dyserythropoiesis were not identified or simply described as non‐regenerative [19].

One of the most compelling associations in this study was between codocytosis severity and hepatobiliary disease. Dogs with higher grades of codocytes (3+ or 4+) had significantly elevated ALT activities and a higher proportion of hepatobiliary diagnoses compared to dogs with 2+ codocytosis. These results support prior literature from both human and veterinary medicine indicating that hepatic dysfunction, particularly involving lipid dysregulation, can alter red blood cell membrane composition and increase membrane surface area, thereby promoting codocyte formation [9, 10, 11, 12]. In humans, codocytes are a recognized feature of liver disease, particularly cholestatic disorders, due to altered lecithin‐cholesterol acyltransferase activity and increased plasma cholesterol levels. In dogs, similar mechanisms have been proposed, and our findings reinforce a hepatocellular contribution to codocyte formation, especially in cases involving congenital hepatic anomalies or gallbladder disease.

Codocytosis was also commonly identified in gastrointestinal or pancreatic disease, particularly in dogs with suspected or confirmed pancreatitis and chronic enteropathies. These diseases may contribute to codocyte development through chronic inflammation, altered lipid absorption, or secondary hepatic effects. Inflammatory bowel disease and protein‐losing enteropathy may impair intestinal lipid handling, leading to downstream effects on erythrocyte membranes, though this mechanism requires further investigation [20]. Interestingly, the strong association between gastrointestinal disease and higher codocyte grades suggests a potential systemic metabolic component that may extend beyond localized pathology.

Diabetes mellitus, especially cases complicated by ketoacidosis, was a notable endocrine comorbidity in this population. This corroborates findings from a recent study by Slead et al., which documented a higher frequency and severity of codocytosis in diabetic dogs, particularly those in diabetic ketoacidosis (DKA) [14]. Hyperlipidemia, oxidative stress, and changes in membrane integrity during DKA may all contribute to this phenomenon. These findings mirror observations in human diabetics, where altered erythrocyte deformability and membrane lipid content have been documented [3].

Neoplastic disease was the most frequently recorded diagnosis overall but did not show a statistically significant association with codocyte severity. It is possible that codocytosis in these cases represents a paraneoplastic phenomenon, secondary to treatment, or is coincidental, given the high prevalence of cancer in the study population and the frequency with which CBCs are performed in dogs undergoing cancer treatment.

Medication use was highly prevalent in this cohort, with over 70% of dogs receiving at least one drug at the time of blood collection. Antibiotics, particularly metronidazole and penicillins, were the most frequently administered, and their use was significantly associated with higher codocyte grades. Although no direct causative link between these medications and codocytosis has been established in veterinary or human literature, their association may reflect the underlying severity or nature of illness in these patients rather than a direct erythrotoxic effect. Notably, prior studies have not implicated antimicrobial agents in codocyte formation, but further investigation may be warranted given this correlation.

Collectively, these findings support a multifactorial pathogenesis for codocytosis in dogs, with hepatobiliary dysfunction, gastrointestinal pathology, and endocrine disease (especially diabetes) all contributing potential mechanistic roles. The absence of associations with anemia or reticulocytosis in most cases indicates that codocytosis is not a simple marker of erythropoietic activity but may instead reflect membrane‐level alterations driven by lipid metabolism, oxidative stress, or impaired splenic clearance.

This study has several limitations, including its retrospective design, the varied diagnostic evaluations, the fact that the population of dogs was exclusively from a specialty hospital, and the reliance on semi‐quantitative blood smear interpretation, which may be subject to interobserver variability. Additionally, only dogs with ≥ 2+ codocytosis were included, potentially excluding cases with subtle but clinically meaningful findings.

## 5 Conclusion

While traditionally associated with anemia, codocytes in this study were more frequently observed in dogs with hepatobiliary, gastrointestinal, and metabolic disorders, particularly those involving lipid metabolism, such as diabetes mellitus and liver disease. These findings suggest that codocytosis may reflect underlying systemic pathology rather than erythropoietic response alone. Recognition of this poikilocyte on blood smear review should prompt clinicians to consider broader metabolic or hepatobiliary evaluation. Further prospective studies are warranted to elucidate the prognostic relevance and pathophysiologic mechanisms underlying codocytosis in canine patients.

## Conflicts of Interest

The authors declare no conflicts of interest.
